# A Rare Case of Concurrent Chronic Lymphocytic Leukemia and Therapy-Unrelated Acute Myeloid Leukemia That Evolved From Myelodysplastic Neoplasm

**DOI:** 10.7759/cureus.108281

**Published:** 2026-05-05

**Authors:** Surbhi Bansil, Steven Y Lai, Amanda E Lo, Robin Dietz, Jarred P Reed

**Affiliations:** 1 Internal Medicine, Olive View-University of California Los Angeles Medical Center, Sylmar, USA; 2 Hematology and Oncology, Olive View-University of California Los Angeles Medical Center, Sylmar, USA; 3 Pathology, Olive View-University of California Los Angeles Medical Center, Sylmar, USA

**Keywords:** acute myeloid leukemia (aml), chromosome 7 deletion, chronic lymphocytic leukemia (cll), hematologic neoplasms, myelodysplastic syndrome (mds), nf1 mutation, runx1 gene

## Abstract

It is unusual for patients to have two or more concurrent hematologic malignancies. Simultaneous chronic lymphocytic leukemia (CLL) and acute myeloid leukemia (AML) are even more rare, and most reported cases are therapy-related. The incidence of cases of concurrent CLL and therapy-unrelated AML is even more rare at <1%. In this case report, we present a very rare case of a patient with concurrent CLL and therapy-unrelated myelodysplastic neoplasm (MDS) that transformed to AML. Peripheral flow cytometry and a bone marrow biopsy showed separate populations of myeloblasts and monoclonal B-lymphocytes. Both populations of cells were subjected to fluorescence in situ hybridization (FISH) testing and next-generation sequencing (NGS) which revealed an abnormal clone with the deletion of the long arm of chromosome seven (7q-) and RUNX1 and NF1 among myeloid cells, suggestive of adverse risk MDS-related AML. These cytogenetic findings were not observed among the lymphoid cells tested. The pathogenesis of concurrent CLL and de novo AML is unknown and requires further investigation. Given the differing cytogenetics among each cell population, the CLL and AML may have developed as separate clonal processes. With a further understanding of the pathogenic mechanism, this may influence the treatment decision-making process.

## Introduction

Chronic lymphocytic leukemia (CLL) is an indolent lymphoproliferative disorder that involves the excessive proliferation and accumulation of dysfunctional monoclonal B-lymphocytes within the blood, bone marrow, and lymphoid tissues. There is variability in the clinical presentation, course, and prognosis of CLL among different patients. Some patients may be asymptomatic, while others may present with lymphadenopathy, anemia and/or thrombocytopenia, constitutional symptoms, hepatomegaly, and/or splenomegaly [1,2]. CLL is staged using the Rai and Binet systems and is prognosticated based on performance status, age, and the presence of cytogenetic or chromosomal abnormalities [1]. Treatment is typically indicated for those with advanced-stage disease as early intervention for early-stage disease has not been shown to impact survival [1,2]. 

In contrast to CLL, acute myeloid leukemia (AML) is an aggressive myeloproliferative neoplasm that involves the excessive clonal expansion and accumulation of myeloblasts within the bone marrow and blood, resulting in anemia and thrombocytopenia [3,4]. There is high variability in clinical presentation, and most commonly, symptoms are related to anemia, such as fatigue, generalized weakness, and dyspnea. Patients can also experience excessive bleeding from thrombocytopenia, recurrent infections from neutropenia, and hepatosplenomegaly [3,4]. Diagnosis requires the presence of ≥20% myeloid blasts within peripheral blood confirmed with flow cytometry, and prognostication depends on age, cytogenetics and chromosomal abnormalities, and treatment response [3,4]. Patients with myelodysplastic neoplasm (MDS), a group of blood disorders characterized by abnormal blood cell production within the bone marrow, can often progress to AML, with an estimated incidence rate of 30% [5]. 

It is very rare for CLL and AML to occur simultaneously, with most reported cases being therapy-related [6-9]. The incidence of cases of concurrent CLL and therapy-unrelated AML is even more rare at <1% [3]. Therapy-related AML compared with de novo AML in patients with CLL has been associated with a significantly worse prognosis overall. One retrospective study found that among 66 patients with concurrent CLL or CLL-like disease with concurrent myeloid neoplasms, patients with therapy-related myeloid neoplasms had shorter overall survival (OS) and a higher rate of adverse cytogenetic abnormalities [10]. 

In this paper, we report a rare case of a patient with CLL and concurrent therapy-unrelated MDS-related AML.

## Case presentation

**Figure 1:**
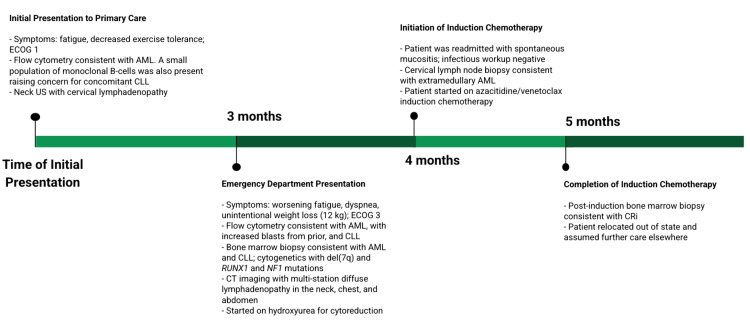
Chronological diagram of events ECOG: Eastern Cooperative Oncology Group; US: ultrasound; AML: acute myeloid leukemia; CLL: chronic lymphocytic leukemia; CT: computed tomography; CRi: complete remission with incomplete count recovery

A 63-year-old man with no known past medical history initially presented to his primary care physician reporting severe fatigue and exercise intolerance, only capable of walking one block before having to stop to rest. His Eastern Cooperative Oncology Group (ECOG) Performance Status was determined to be 1, as he was still ambulatory and capable of self-care and light duties. His physical examination revealed skin pallor and splenomegaly. His family history was unremarkable, with no history of malignancies or blood disorders. The patient was unemployed and had no relevant environmental or occupational exposures. Complete blood count (CBC) showed a leukocytosis with blasts and lymphocytes on the manual differential, as well as a macrocytic anemia and thrombocytopenia (Table 1). Peripheral flow cytometry revealed the presence of myeloblasts accounting for 52% of cells, which was consistent with AML. Interestingly, flow cytometry coincidentally revealed a separate population of CD5-positive/CD23-positive kappa-restricted B-lymphocytes. Prominent cervical lymphadenopathy, with an enlarged left level 3 cervical lymph node measuring 2.1×1.1 cm, was seen on an ultrasound of the neck done earlier that month. Based on the absolute lymphocyte count, which we later found was sustained for at least three months (Table 1, Figure 1), and these additional clinical findings, there was concern for concurrent CLL. Hematology was consulted, and the patient was advised to immediately present to the hospital for further workup and treatment. However, he declined and was then lost to follow-up (Figure 1).

**Table 1:** Summary of our patient's significant laboratory findings

Laboratory test	At the time of the initial presentation	Three months after the initial presentation	Reference range
White blood count (WBC) (cells/L)	27×10⁹	147.1×10⁹	4.5-10×10⁹
Percentage of peripheral blast cells (%)	43%	56%	<5%
Percentage of peripheral lymphocytes (%)	34%	33%	20-40%
Hemoglobin (g/dL)	4.4	2.8	13.5-16.5
Mean corpuscular volume (MCV) (fL)	104.5	113	82-97
Platelet count (cells/L)	65×10⁹	77×10⁹	160-360×10⁹
Prothrombin time (PT) (seconds)	Not available	19.3	11.7-14.2
Partial thromboplastin time (PTT) (seconds)	Not available	36.3	23.5-35.6
International normalized ratio (INR)	Not available	1.66	0.88-1.13
Fibrinogen (mg/dL)	Not available	268	215-450
Blood urea nitrogen (BUN) (mg/dL)	16	16	8-20
Creatinine (mg/L)	1	1.05	0.5-1.2
Potassium (mmol/L)	4.7	4.5	3.6-5.1
Calcium (mg/dL)	8.7	8.2	8.9-10.3
Alkaline phosphatase (U/L)	133	195	38-126
Alanine transaminase (ALT) (U/L)	14	76	15-41
Aspartate aminotransaminase (AST) (U/L)	21	212	14-54
Total bilirubin (mg/dL)	0.4	0.6	0.1-1.2
Serum uric acid (mg/dL)	Not available	8.4	2.6-8
Lactate dehydrogenase (U/L)	Not available	398	98-192
C-reactive protein (mg/L)	6.5	9.3	0-7
Percentage of myeloblasts on peripheral flow cytometry (%)	52%	59%	<5%
Percentage of lymphocytes on peripheral flow cytometry (%)	15.7%	6.8%	20-40%
Absolute lymphocyte count (cells/L)	9.18×10⁹	48.5×10⁹	1-4.8×10⁹

**Figure 2:**
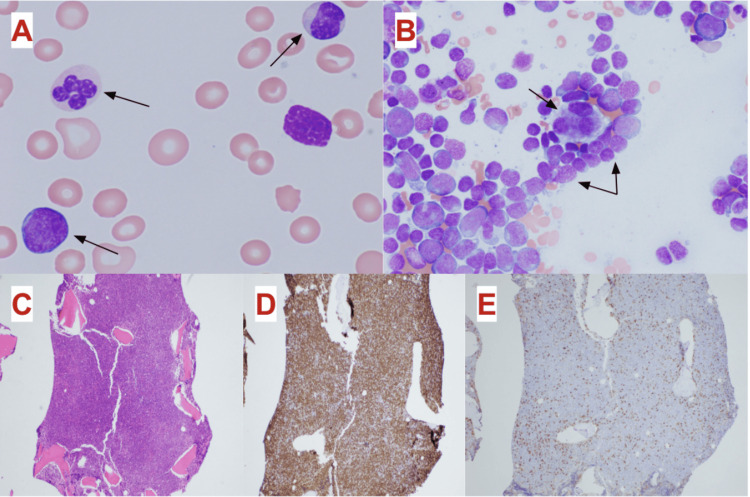
Peripheral blood and bone marrow histologic findings demonstrating cell populations consistent with AML and CLL (A) Peripheral blood smear: myeloblast (bottom left arrow) and hypogranular and mononuclear neutrophils (top left and top right arrows) (original magnification ×400)​. (B) Bone marrow biopsy aspirate: megakaryocyte with nuclear lobe separation (center arrow) surrounded by myeloblasts (hematoxylin and eosin stain; original magnification ×400). (C) Bone marrow biopsy core specimen: hypercellular bone marrow aspirate with sheets of myeloblasts (hematoxylin and eosin stain; original magnification ×20). (D) Bone marrow biopsy core specimen: 80% CD34 positivity among myeloblasts (stained brown) (CD34 immunostain; original magnification ×20). (E) Bone marrow biopsy core specimen: CD20-negative myeloblasts and CD20-positive lymphocytes (stained brown) (CD20 immunostain; original magnification ×20). AML: acute myeloid leukemia; CLL: chronic lymphocytic leukemia

Three months later, the patient presented to the emergency department with worsening fatigue and dyspnea on exertion, generalized weakness, and unintentional weight loss of 13 kilograms over six months. He was now only able to walk a few steps before having to stop to rest. His physical examination remained unremarkable. His ECOG score was now 3, as the patient reported capabilities of limited self-care due to fatigue and requiring confinement to a bed for more than 50% of the day (Figure 1). His CBC showed worsening leukocytosis and macrocytic anemia, as well as persistent thrombocytopenia (Table 1). Reassuringly, he did not meet Cairo-Bishop criteria for tumor lysis syndrome. A peripheral smear showed large lymphocyte predominance and some blasts present (Figure 2). Repeat flow cytometry of peripheral blood re-demonstrated 59% myeloblasts, as well as 6.8% monoclonal B-cells that were seen three months prior (Table 1).

The patient underwent a bone marrow biopsy which revealed a myeloid cell population and a monoclonal B-lymphocyte population. The marrow sample was hypercellular (approximate cellularity of >90%) with reduced multilineage maturation and myelodysplasia; a quantitative differential across all lineages and fibrosis assessment were not done. The myeloid cells were consistent with myeloblasts, estimated to account for >85% of all cells based on CD34 positivity using immunohistochemistry and flow cytometry among both trephine and aspirate samples, 5% granulocytic cells, 1% lymphocytoid cells, and 7% monocytoid cells. The small population of monoclonal B-lymphocytes accounted for 7.2% of lymphoid cells and <0.1% of total cells seen. The immunophenotype of these myeloblasts and lymphocytes is shown in Table 2. Chromosomal analysis showed an abnormal clone with a deletion in the long arm of chromosome seven (7q-), with a karyotype of 46,XY,del(7)(q22q34). Fluorescence in situ hybridization (FISH) testing for BCR::ABL1 fusion t(9;22) was negative. TP53 analysis was negative. Mutations in RUNX1 and NF1 were also identified, with variant allele frequencies (VAF) of 49.2% and 61.8%, respectively, suggestive of germline mutations per Pathology, though confirmatory germline testing was not performed. All of these findings supported adverse-risk MDS-related AML.

**Table 2:** Summary of our patient's immunophenotype findings Positive is indicative of the presence of immunostaining for the specified marker. Negative is indicative of the absence of immunostaining for the specified marker. TdT: terminal deoxynucleotidyl transferase; HLA-DR: human leukocyte antigen-DR isotype; MPO: myeloperoxidase

Cluster of differentiation	Myeloblasts	Lymphocytes
CD5	Negative	Positive
CD7	Negative	Negative
CD10	Negative	Negative
CD11c	Dim positive	Negative
CD13	Moderately positive	Negative
CD19	Negative	Positive
CD20	Negative	Positive
CD23	Negative	Negative
CD33	Dim positive	Negative
CD34	Moderately positive	Negative
CD38	Moderately positive	Negative
CD117	Moderately positive	Negative
cCD79a	Negative	Negative
cCD3	Negative	Negative
TdT	Negative	Negative
HLA-DR	Negative	Negative
MPO	Dim positive	Negative
Kappa light chain	Negative	Positive

Computed tomography (CT) scans of the patient's neck, chest, abdomen, and pelvis revealed extensive lymphadenopathy. In comparison with a CT scan done eight months prior, there were progressive hepatomegaly measuring 18.5 cm (from 10 cm) and worsening splenomegaly measuring 19.1 cm (from 13.1 cm). Magnetic resonance imaging (MRI) of the brain did not show any evidence of central nervous system (CNS) involvement. The patient was started on hydroxyurea 3 grams twice daily for cytoreduction, as well as allopurinol 300 mg twice daily to prevent tumor lysis syndrome (Figure 1). With regard to antimicrobial prophylaxis, we started acyclovir 400 mg twice daily and isavuconazole 372 mg once daily for infectious prophylaxis. His leukocytosis improved to 45,000 cells/µL, and he was discharged six days later. 

**Figure 3:**
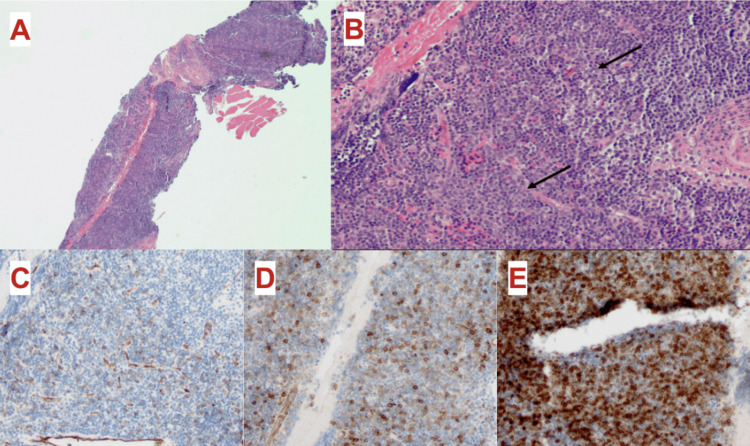
Cervical lymph node biopsy histologic findings consistent with extramedullary AML (A) Lymph node core biopsy: with effacement of nodal architecture (hematoxylin and eosin stain; original magnification ×20). (B) ​Lymph node core biopsy: foci (arrows) of mononuclear cells with open chromatin​ (hematoxylin and eosin stain; original magnification ×100). (C) Lymph node core biopsy: weak positive immunostaining for CD34 highlights blasts in the lymph node (CD34 immunostain; original magnification ×100). (D) Lymph node core biopsy: CD117 immunostaining highlights myeloblasts in the lymph node (CD117 immunostain; original magnification ×100). (E) Lymph node core biopsy: CD10 immunostaining demonstrates excess myeloid mononuclear cells in the lymph node, consistent with myeloblasts (CD10 immunostain; original magnification ×100)​. AML: acute myeloid leukemia

After discharge, the patient was planned for close follow-up with hematology for the initiation of treatment for AML. Unfortunately, he started to develop symptoms of spontaneous mucositis with severe oropharyngeal pain and tongue swelling and was again re-admitted to the hospital (Figure 1). A broad infectious workup with blood cultures and herpes simplex virus culture testing was unremarkable. A repeat CT of the neck revealed mild pharyngeal wall edema adjacent to one of the oral lesions, but no evidence of abscess or other deep tissue infection. During this admission, he underwent a biopsy of a cervical lymph node to rule out other lymphoproliferative processes. Histology revealed a population of myeloblasts, consistent with extramedullary infiltration of AML (Figure 3). He was then started on induction chemotherapy with a combination of azacitidine (75 mg/m^2^ on days 1-7) and venetoclax (50 mg on day 1, 50 mg on day 2, 100 mg on day 3, 200 mg on days 4-28), which was chosen based on his age and adverse-risk AML [11]. The decision was made to forgo treatment of the patient's likely indolent CLL given its CD38-negative phenotype and pursue the treatment of AML given the aggressive nature of the disease. Hydroxyurea was discontinued when induction chemotherapy was initiated. Notably on day 1 of therapy, the patient was neutropenic with an absolute neutrophil count of 0. Antimicrobial prophylactic coverage with levofloxacin 500 mg once daily was started. On day 2 of therapy, the serum uric acid level increased to >10 mg/dL. A one-time dose of rasburicase at 0.2 mg/kg was given. He was provided supportive transfusions with a total of six units of irradiated packed red blood cells to maintain a Hgb >7 g/dL. No platelet or plasma transfusions were needed. By day 10, the patient had recovery in neutropenia and stabilization of his other cytopenias.

On day 31, a bone marrow biopsy following the completion of induction therapy showed hypocellularity with megakaryocytic and myeloid atypia. This was consistent with a complete remission with incomplete count recovery (CRi), with 1% blasts detected and marked pancytopenia (white blood count (WBC) 0.8×10⁹cells/L, hemoglobin 6.8 g/dL, platelet 82×10⁹ cells/L). FISH, flow cytometry, karyotype analysis, and next-generation sequencing showed persistent cytogenetic abnormalities: del(7q) and RUNX1 and NF1 mutations. Given the high suspicion for germline mutations, our team recommended that the patient and his family pursue genetic counseling. Further treatment response could not be monitored as the patient moved to a different state following the completion of induction therapy and assumed further care elsewhere (Figure 1).

## Discussion

This patient was diagnosed with MDS-related AML and concurrent CLL. The presence of >20% myeloblasts seen in the patient's bone marrow met the criteria for AML. In addition, his elevated monoclonal B-lymphocyte population of ≥5×10⁹ cells/L, extensive lymphadenopathy, and hepatosplenomegaly were consistent with CLL. 

Interestingly, although CLL is typically associated with lymphadenopathy and splenomegaly, the biopsy of his lymph node only showed AML without CLL. This was possibly due to the more aggressive nature of AML or sampling error. Based on the findings of RUNX1 and NF1 mutations, we suspect that the underlying MDS transformed into AML. Due to the high allelic frequencies of the mutations, they were suspected to be germline mutations which predisposed this patient to develop AML [12]. 

A RUNX1 mutation can be seen in up to 10-16% of AML cases as well as 12-24% of MDS cases [13-15]. Inherited RUNX1 mutations are inherited in an autosomal-dominant pattern which can result in a condition called familial platelet disorder (FPD), which predisposes patients to developing myeloid malignancies such as AML [13-15]. Similarly, NF1 mutations are also commonly identified in such patients, with about 5-10% of cases of MDS and 10-15% of cases of AML [16]. Germline mutations in NF1 classically are associated with the autosomal-dominant inherited disorder called neurofibromatosis type 1, which in itself is referred to as a cancer predisposition syndrome; however, not infrequently have somatic mutations been identified among AML cases [16]. Patients with MDS who harbor RUNX1 and/or NF1 mutations should receive both genetic and family counseling as both abnormalities can predispose patients to hematologic disorders and neoplasms. Our literature search did not reveal an association between these genes and CLL. Furthermore, pathogenic complete or partial deletions of chromosome 7 have also been implicated in predisposing patients with MDS to transformation to AML, which was also identified in our patient [17].

CML frequently transforms into either AML or ALL with an incidence of 10-20% [18]. However, the incidence of transformation of CLL to acute leukemia (either AML or ALL) is reportedly less than 1% [1,2]. Most reported cases of patients with CLL who later developed AML were in the setting of treatment for CLL, most notably with purine analogs and alkylating agents [6-9]. These cases of therapy-related AML arose on average 3-6 years following CLL therapy. 

Literature review shows very few patients with therapy-unrelated concurrent CLL and AML [3,19-32]. Table 3 summarizes the clinicopathologic characteristics of these rare cases. Notably, a larger-scale study by Robertson et al. showed that among 1374 patients at MD Anderson Cancer Center who had a history of CLL, only two patients were found to have concurrent diagnoses of CLL and AML/MDS without having undergone CLL-related therapy [9].

**Table 3:** Comparison of our patient with cases of therapy-unrelated CLL with MDS or AML AML: acute myeloid leukemia; CLL: chronic lymphocytic leukemia; MDS: myelodysplastic neoplasm

Study	Patient age (years)/sex (M or F)	Diagnosis	Immunohistochemistry		Cytogenetics
			Blast cells	B-lymphocytes	
Our patient	63/M	CLL/AML	CD13+, CD33+, CD34+, CD38+, CD117+, TdT+, HLA-DR+	CD5+, CD19+, CD20+, Kappa+	46,XY,del(7)(q22q34)
Conlan and Mosher (1989) [22]	69/M	CLL/AML	Not available	Not available	Not available
Lima et al. (1996) [32]	66/F	CLL/AML (M2)	CD13+, CD33+, CD34+, CD38+, CD71+	CD5+. CD19+, HLA-DR+, IgM, Kappa+	43,XX,t(3;12)(q23;p13),del(5)(q14),- 7,+add(12)(p13), -13, -15, i(21)(q10),der(21;22)(q10;q10)
Sylvester et al. (1997) [23]	85/F	CLL/MDS	Not available	CD5+, CD20+, CD19+, CD23+, HLA-DR+, IgM, IgD	46,XX,del(13)(?q12q22); trisomy 12
Mateu et al. (1997) [24]	59/M	CLL/AML (M1)	CD4 dim+, CD13+, CD33+, CD56+, cMPO+	CD19+, CD20+, CD21+, CD5+, CD23+, CD43+	Non-clonal chromosomal abnormalities: del(7)(q22); t(1;4)(p36;q26); t(6;7)(p23;p15); t(1;9)(q25;p23)
Lai et al. (1999) [3]	57/M	CLL/AML (M1)	CD13+, CD15+, CD33+, MPO+, Sudan black B+	CD5+, CD19+, HLA-DR+, Kappa+	Not available
	78/M	CLL/AML (M0)	CD24+, CD45+, CD117+, HLA-DR+, CD43+	CD5+, CD19+, CD23+, IgM, Kappa+	Not available
	87/M	CLL/AML (M5)	CD11c+, CD13+, CD33+, CD34+, CD45+, c-kit+, HLA-DR+, ANBE+	CD11c+, CD19+, CD20+, Kappa+, IgG	No chromosomal abnormalities
	80/M	CLL/AML (M2)	MPO+	CD20+, CD43+	Not available
	67/M	CLL/MDS	N/A	CD5+, CD19+, CD20+, CD43+,	Not available
Ornellas de Souza et al. (2001) [25]	70/F	CLL/AML (M2)	HLA-Dr+, CD13+, CD33+, CD34+	CD19+, CD5+, HLA-DR+, weak SMIg+, Kappa+	47,XX, +12(10)/46,XX, del(5)(q31),t(8;13)(q22;q21)(4)/46, XX(6)
Muta et al. (2002) [26]	84/F	CLL/AML (M2)	CD13+, CD33+, CD34+, HLA-DR+	CD5+, CD19+, CD20+, lambda+	56,XY,+1,+6,+8,add(10)(q26),+11,+11,+13, +14,+15,+21,+21(19)/46,XY(1)
Gottardi et al. (2006) [27]	69/M	CLL/AML (M2)	CD34+, CD13+, CD33+, HLA-DR+, CD7+	CD19+, CD5+, CD23+	No chromosomal abnormalities
Hatoum et al. (2007) [20]	66/M	CLL/AML (M4)	CD13+, CD33+, CD34+, CD11c+, HLA-DR+, CD14+	CD5+, CD19+, CD20+, CD22+, CD23+, HLA-DR+	T-cell receptor gamma gene rearrangement
Zhang et al. (2011) [28]	80/M	CLL/AML (M0)	CD13+, CD33+, CD115 partial+, CD117+, CD34+, HLA-DR+, CD7 dim+	CD5+, CD10+, CD20 dim+, CD22+, CD23+, kappa dim+	46,XY,t(2;5)(q37;q31)(19)/46,XY(1)
Cazaceanu et al. (2012) [19]	61/M	CLL/AML (M6)	CD13+, CD34+, CD117+, CD61+, Glycophorin A+, CD45 dim+	CD19+, CD20+, CD5+, CD23+, CD79b low+, CD38-, FMC7-, ZAP70+	Not available
Al Mussaed et al. (2016) [28]	77/M	CLL/AML	CD13+, CD33+, CD14+, CD4+, CD15+, CD64+, HLA-DR+, CD11c+	CD19+, CD5+, CD23+, CD20+	No chromosomal abnormalities
Lee et al. (2017) [21]	76/M	CLL/AML	CD34+, CD13+, CD33+, CD117+, and HLA-DR+	CD5+, CD19+, CD20+, CD23+	46,XY,del(13)(q14),add(14)(q32)[3]/46,XY(17); Del 13
Shoyele and Gupta (2018) [31]	65/M	CLL/AML (M5)	CD24+, cMPO+, CD117+, HLA-DR+, CD64+, CD13+, CD33 dim+, CD15 dim+	CD19+, CD20+, CD5+, CD23+	inv(16)(p13q22) CBFB-MYH11; trisomy 8; monosomy 18; trisomy 12; RUNX1T1 mutation; trisomy 12, RUNX1 mut, inv(16)(p13;q22)
Bhar et al. (2018) [30]	85/M	CLL/AML	CD33+, CD64+, CD14+, HLA-DR+, CD13 dim+	CD19+, cyDC79a+, CD20 dim+, CD5+, CD23+, CD200+, Kappa dim+	Not available

The pathogenesis of coexisting CLL and therapy-unrelated MDS or AML is unknown. Several hypotheses have been discussed. In general, patients with CLL have an increased risk of developing second malignancies with an incidence of 9-20%, typically solid organ neoplasms of the colon and lung, sarcomas, and skin cancer [3]. On the other hand, the risk of developing a second hematologic malignancy among these patients is unusual. Cases of concurrent CLL and multiple myeloma have been cited among the pool of cases of CLL and second hematologic malignancies, which was likely due to the expansion of the same B-cell clone [33]. 

Some hypothesize that a similar phenomenon may explain the simultaneous occurrence of CLL with MDS or AML, in which a common clonal precursor may have later differentiated into two different lineages. Lima et al. found that in a patient with concurrent CLL and AML, similar chromosomal abnormalities were seen between the CLL and AML populations (i.e., chromosome 5 deletion, monosomy 7, and trisomy 12), which led them to believe that both malignancies arose from the same clone [32]. However, it is unusual for cells in CLL to undergo spontaneous mitosis. Therefore, it can be argued that the karyotype observed only reflected the myeloblasts. A study reported a case of concurrent AML and CLL in which the chromosomal abnormalities add(14q) and del(13) were also observed among both myeloid and lymphoid lineages, suggestive of a common progenitor clonal origin [21].

Others support a contrary view to the single-clone hypothesis and theorize that both malignancies arise from different clones. Gottardi et al. described a patient with concurrent CLL and AML who was found to have an IgH gene arrangement among their B-lymphocyte population (CD19+/CD34-), which was not observed in their myeloid population (CD19-/CD34+), suggestive of two separate clonal processes [27]. Another study of concurrent CLL and MDS found that both the myeloid population and the lymphoid population had different chromosomal abnormalities, with the lymphoid cells having trisomy 12, while the myeloid cells had a 13q- deletion; this also supported the theory of separate clonal disorders [23]. In the case of our patient, the 7q deletion was only observed among myeloid cells and not lymphoid cells, which may support the hypothesis that CLL and AML developed as separate clonal processes.

Decision-making for treating patients with concurrent CLL and AML is complex. CLL causes cumulative immunosuppression via innate and adaptive immune dysfunction, such as chronic hypogammaglobulinemia and T-cell dysfunction, resulting in increased susceptibility to infection [34]. This can be risky with intensive AML-directed treatment regimens, and aggressive infectious prophylaxis for these patients is required. In the case of our patient, given the indolent nature of CLL and the aggressive, rapidly progressive nature of AML with high-risk cytogenetic features, treatment of AML took precedence of CLL and was tailored towards the patient's age, performance status, and disease risk. The patient was started on appropriate antimicrobials for infectious prophylaxis. 

Allogeneic hematopoietic stem cell transplantation (allo-HSCT) may be considered as well in patients with these concurrent malignancies, though outcomes are associated with high morbidity and mortality. For CLL, allo-HSCT can provide long-term disease control but can be associated with significant non-relapse mortality (NRM). Bennett et al. report that 62 patients with CLL who underwent allo-HSCT from 2000 to 2022 had modest outcomes with a median two-year OS rate of 74%, a progression-free survival (PFS) rate of 57%, and an NRM rate of 18% [35]. In contrast, patients with treatment-refractory or relapsed AML had worse outcomes. Azevedo et al. found that among 28 patients who underwent allo-HSCT from 2010 to 2024, the median one-year OS rate was 46%, the PFS rate was 43.5%, and the NRM rate was 32% [36]. Patients with both CLL and AML may suffer an even more significantly worse outcome after allo-HSCT as demonstrated by a multicenter retroactive registry study of patients with secondary AML with antecedent lymphoid malignancies (including CLL), which found that the median two-year OS rate was 37%, the leukemia-free survival rate was 32%, and the NRM rate was 29% [37].

## Conclusions

We have presented a very rare case of a patient with de novo MDS-related AML and concurrent CLL. The pathogenesis of concurrent CLL and de novo AML is largely unknown. In our patient, there were molecular differences between the myeloid and lymphoid cell populations, such as the deletion of the long arm of chromosome seven (7q-) and RUNX1 and NF1 mutations, which may favor a previously suggested hypothesis that these malignancies may have arisen from separate clonal processes. The decision-making process for the treatment of patients with concurrent CLL and AML is clinically challenging, given the high risk of infection and refractory disease and limited benefit from advanced treatment options such as allo-HSCT. Further investigations may be helpful for influencing the treatment decision-making process.

## References

[1] Byrd JC, Stilgenbauer S, Flinn IW (2004). Chronic lymphocytic leukemia. Hematology Am Soc Hematol Educ Program.

[2] Mukkamalla SKR, Taneja A, Malipeddi D, Master SR (2023). Chronic lymphocytic leukemia. StatPearls [Internet].

[3] Lai R, Arber DA, Brynes RK, Chan O, Chang KL (1999). Untreated chronic lymphocytic leukemia concurrent with or followed by acute myelogenous leukemia or myelodysplastic syndrome: a report of five cases and review of the literature. Am J Clin Pathol.

[4] Döhner H, Wei AH, Appelbaum FR (2022). Diagnosis and management of AML in adults: 2022 recommendations from an international expert panel on behalf of the ELN. Blood.

[5] Menssen AJ, Walter MJ (2020). Genetics of progression from MDS to secondary leukemia. Blood.

[6] Morrison VA, Rai KR, Peterson BL (2002). Therapy-related myeloid leukemias are observed in patients with chronic lymphocytic leukemia after treatment with fludarabine and chlorambucil: results of an intergroup study, cancer and leukemia group B 9011. J Clin Oncol.

[7] Wu Y, Liu S, Wang D, Yao X (2023). Acute myeloid leukemia secondary to chronic lymphocytic leukemia after prolonged chlorambucil therapy: a case report. Pharmgenomics Pers Med.

[8] Benjamini O, Jain P, Trinh L (2015). Second cancers in patients with chronic lymphocytic leukemia who received frontline fludarabine, cyclophosphamide and rituximab therapy: distribution and clinical outcomes. Leuk Lymphoma.

[9] Giri S, Bhatt VR, Khanal S, Ganti AK (2015). Treatment-related acute myeloid leukemia in a chronic lymphocytic leukemia patient: role of fludarabine?. Ther Adv Hematol.

[10] Luedke C, Zhao Y, McCracken J (2022). Myeloid neoplasms in the setting of chronic lymphocytic leukaemia/chronic lymphocytic leukaemia-like disease: a clinicopathological study of 66 cases comparing cases with prior history of treatment to those without. J Clin Pathol.

[11] DiNardo CD, Pratz K, Pullarkat V (2019). Venetoclax combined with decitabine or azacitidine in treatment-naive, elderly patients with acute myeloid leukemia. Blood.

[12] Kennedy AL, Shimamura A (2019). Genetic predisposition to MDS: clinical features and clonal evolution. Blood.

[13] Gaidzik VI, Bullinger L, Schlenk RF (2011). RUNX1 mutations in acute myeloid leukemia: results from a comprehensive genetic and clinical analysis from the AML study group. J Clin Oncol.

[14] Simon L, Spinella JF, Yao CY (2020). High frequency of germline RUNX1 mutations in patients with RUNX1-mutated AML. Blood.

[15] Khan M, Cortes J, Kadia T (2017). Clinical outcomes and co-occurring mutations in patients with RUNX1-mutated acute myeloid leukemia. Int J Mol Sci.

[16] Eisfeld AK, Kohlschmidt J, Mrózek K (2018). NF1 mutations are recurrent in adult acute myeloid leukemia and confer poor outcome. Leukemia.

[17] Mori M, Kubota Y, Durmaz A (2023). Genomics of deletion 7 and 7q in myeloid neoplasm: from pathogenic culprits to potential synthetic lethal therapeutic targets. Leukemia.

[18] Copland M (2022). Treatment of blast phase chronic myeloid leukaemia: a rare and challenging entity. Br J Haematol.

[19] Cazaceanu O, Iova A, Vladareanu AM, Onisai M, Enache C, Andrus E (2012). Chronic lymphocytic leukemia followed by myelodysplastic syndrome and erythroleukemia. J Med Life.

[20] Hatoum HA, Mahfouz RA, Otrock ZK, Hudaib AR, Taher AT, Shamseddine AI (2007). Acute myeloid leukemia with T-cell receptor gamma gene rearrangement occurring in a patient with chronic lymphocytic leukemia: a case report. Am J Hematol.

[21] Lee HY, Park CJ, You E, Cho YU, Jang S, Seo EJ (2017). A case of acute myeloid leukemia concurrent with untreated chronic lymphocytic leukemia. Ann Lab Med.

[22] Conlan MG, Mosher DF (1989). Concomitant chronic lymphocytic leukemia, acute myeloid leukemia, and thrombosis with protein C deficiency. Case report and review of the literature. Cancer.

[23] Sylvester LS, Nowell PC, Bonner H, Moreau L, Moore JS (1997). Concurrent diagnosis of chronic lymphocytic leukemia and myelodysplastic syndrome. Leuk Res.

[24] Mateu R, Bellido M, Sureda A, González Y, Rubiol E, Aventin A, Nomdedéu J (1997). Concomitant chronic lymphocytic leukemia and acute myeloid leukemia with an uncommon immunophenotype. Am J Hematol.

[25] Ornellas De Souza MH, de Souza Fernandez T, Diamond HR, Maioli MC, Pitanga Bacha PC, De Lucena SB (2001). Cytogenetic and immunophenotypic evidence of independent clonal origins of concomitant chronic lymphocytic leukaemia and acute myeloid leukaemia. Eur J Haematol.

[26] Muta T, Okamura T, Niho Y (2002). Acute myelogenous leukemia concurrent with untreated chronic lymphocytic leukemia. Int J Hematol.

[27] Gottardi M, Gattei V, Degan M (2006). Concomitant chronic lymphocytic leukemia and acute myeloid leukemia: evidence of simultaneous expansion of two independent clones. Leuk Lymphoma.

[28] Zhang R, Kim YM, Lu X (2011). Characterization of a novel t(2;5;11) in a patient with concurrent AML and CLL: a case report and literature review. Cancer Genet.

[29] Al Mussaed E, Osman H, Elyamany G (2016). Simultaneous existence of acute myeloid leukemia and chronic lymphocytic leukemia: a case report. BMC Cancer.

[30] Bhar VS, Gupta V, Sharma M, Dhawan R, Modi S, Vijyaran M (2018). Rare coexistence of acute monoblastic leukemia with chronic lymphocytic leukemia. Case Rep Hematol.

[31] Shoyele O, Gupta G (2018). Synchronous diagnosis of de novo acute myeloid leukemia with inv(16)(p13q22) and chronic lymphocytic leukemia: a case report and review of the literature. Ann Clin Lab Sci.

[32] Lima M, Porto B, Rodrigues M (1996). Cytogenetic findings in a patient presenting simultaneously with chronic lymphocytic leukemia and acute myeloid leukemia. Cancer Genet Cytogenet.

[33] Aktan M, Akkaya A, Doğan O, Dincol G (2003). Chronic lymphocytic leukemia and multiple myeloma in the same patient: case report. Leuk Lymphoma.

[34] Dearden C (2008). Disease-specific complications of chronic lymphocytic leukemia. Hematology Am Soc Hematol Educ Program.

[35] Bennett R, Frawley T, Thompson PA (2025). A single-institution study of allograft outcomes for chronic lymphocytic leukaemia over 20 years. Intern Med J.

[36] Azevedo FS, Campos V, Szor RS (2024). Impact of allogeneic hematopoietic stem cell transplantation on acute myeloid leukemia patients without complete remission. Blood.

[37] Gatwood KS, Labopin M, Savani BN (2020). Transplant outcomes for patients with therapy-related acute myeloid leukemia with prior lymphoid malignancy: an ALWP of EBMT study. Bone Marrow Transplant.

